# Caregiving intensity and its association with subjective views of ageing among informal caregivers with different sociodemographic background: a longitudinal analysis from Germany

**DOI:** 10.1007/s10433-023-00797-4

**Published:** 2024-01-13

**Authors:** Larissa Zwar, Hans-Helmut König, André Hajek

**Affiliations:** Department of Health Economics and Health Services Research, University Medical Center Hamburg-Eppendorf, 20246 Hamburg, Germany

**Keywords:** Informal caregivers, Subjective age, Attitudes towards own ageing, Philadelphia Geriatric Center Morale Scale, Burden, Care intensity, Care tasks, Gender differences, Age differences, Longitudinal, Panel analysis

## Abstract

We analysed whether care time, burden and range of caregiving tasks were associated with informal caregivers’ subjective views of ageing (measured as attitudes towards own age (ATOA), subjective age (SA), and onset of old age (OOA)), and whether these associations differed as a function of the caregivers’ age and gender. Adjusted cluster-robust fixed effects regression analyses were conducted with gender and age as moderators using data of informal caregivers (≥ 40 years) of the population-based German Ageing Survey (2014, 2017). All three aspect of care intensity were associated with changes in subjective views of ageing and this pattern was a function of the caregiver’s age and gender. Care time was significantly associated with higher SA. Care tasks were significantly associated with more positive ATOA and earlier OOA. Age moderated the association between burden and ATOA, with older adults reporting more positive ATOA. Gender moderated the association between care time and ATOA; women reported less positive ATOA than men with increasing care time, but also felt subjectively younger than men with a broader range of care tasks. Age- and gender-stratified analysis indicated further differences. Our findings suggest to reduce care time, especially among older and female caregivers, to prevent a worsening of views of ageing, while being involved in a broad range of care tasks seems to (only) benefit female caregivers.

**Supplementary Information:**

The online version contains supplementary material available at 10.1007/s10433-023-00797-4.

## Background

### Subjective views of ageing and informal caregiving

Subjective views of ageing can include perceptions of age in general or of one’s own ageing process (Chasteen and Cary 2015; Wurm and Westerhof 2015). In this study, we analyse *attitudes towards one’s own ageing* (ATOA) and *subjective age* (SA) as indicators of views of one’s own ageing process, i.e. personal views. Both measure different aspects of views of ageing, but are related with each other (Bodner et al. 2017; Diehl et al. 2014). SA is active on a conscious level while ATOA is already active on a sub- and preconscious level and also includes affective, cognitive and evaluative factors which reflect internalized societal as well as individual attitudes (Diehl et al. 2014; Hess 2006). *Onset of old age* (OOA) was included as indicator of views of age in general as an addition to the aforementioned personal views (Shrira et al. 2022).

A worsening of these views of ageing can impact health, well-being, and longevity negatively, while more positive views of ageing can be beneficial to these outcomes (Alonso Debreczeni and Bailey 2020; Chang et al. 2020; Kotter-Gruhn et al. 2009; Westerhof et al. 2014, 2023). Thus, better, respective, improving views of ageing are of relevance to informal caregivers, that is, to relatives or friends providing unpaid support to individuals with care needs, who often report worse health and well-being due to their care performance (Bom et al. 2019; Zwar et al. 2018).

Only very few studies have analysed the association between informal caregiving and views of ageing and findings point in both directions, improvement and worsening of views of ageing. For example, one study pointed towards a worsening in the attitude towards older adults among caregivers (Luchesi et al. 2016), while another study indicated more positive attitudes towards ageing among caregivers (Loi et al. 2015). A worsening of views of ageing is in line with *terror management theory applied to ageing* (TMT-A; Martens et al. 2005). The new experiences and increased confrontation with impairments and dependency could remind caregivers of their own vulnerability and mortality, showing them a fate they may share eventually. This can result in distress, disgust and wishing to avoid or devalue these reminders (i.e. negative subjective views of ageing) and activate their negative age views. However, reminders of mortality may also change goals, as *socioemotional selectivity theory* indicates (SST, Carstensen et al. 1999; Löckenhoff and Carstensen 2004). People who perceive themselves closer to death, focus more on emotionally fulfilling and meaningful goals, such as improving relationships and feeling valued. This is in line with the findings of our own previous work. In the previous study we analysed onset and end of caregiving and found that these are associated differently with views of ageing in the group of caregivers aged ≥ 80 years (Zwar et al. 2022). Older caregivers benefited at the onset in terms of better views of ageing (more positive ATOA) but not at the end of care (higher SA). Thus, caregiving may have emphasized more positive aspects of caregiving and fulfilled more emotionally meaningful goals.

In sum, previous research already points to an association between informal caregiving and changes of views of ageing; however, more research is still needed to understand these mechanisms. It remains unclear which aspects of the caregiving performance are relevant to the different indicators of views of ageing. Therefore, we intend to build on and expand our previous work with this study, in which we aim to identify aspects of the care situation, which motivate changes in views of ageing among caregivers. We assume that intensity of informal caregiving in particular is of importance.

So far, very few studies have focused on specific aspect of care and their association with views of ageing. First findings indicated that lower caregiving burden is associated with lower anxiety of ageing (Hamama-Raz et al. 2022). An effect of the burden of caregiving among adult children on the views of ageing of their care-receiving parents was found, although in these dyadic analyses burden was not associated with perceptions of own ageing among the caregiving adult children (Kim et al. 2023). More research is therefore needed that analyses changes in views of ageing in association with different aspects of caregiving intensity. Our findings will highlight how caregiving could be designed to support positive or at least prevent negative views of ageing and could be very helpful due to the relevance of views of ageing for health and well-being (Tully-Wilson et al. 2021; Westerhof et al. 2023).

### The role of care intensity for views of ageing

Higher intensity of caregiving is associated with worse health and psychosocial well-being (Bremer et al. 2015). In terms of more care hours and tasks, it is usually associated with more support needs (Rodríguez-González et al. 2021). Thus, higher care intensity provides more opportunity for confrontation with dependency and illness, and reminders of mortality. Moreover, higher care intensity may activate more age-related stereotypes, such as relating exhaustion or tiredness due to caregiving to age. Therefore, we expect higher intensity to be a relevant predictor for changes in views of ageing.

We aim to analyse different indicators of caregiving intensity, namely hours of care per week, range of care tasks and burden of care. While these factors are related, they focus on different aspects of intensity. The range of care tasks indicates the diversity in care provision and thus is more a qualitative aspect of intensity, while caregiving time is more of a quantitative indicator. Both are also objective indicators of care intensity. A subjective indicator is caregiver burden. Burden reflects the level of care-specific stress and provides insight into the subjective perception of care intensity (Graessel et al. 2014), with which it is associated (Rodríguez-González et al. 2021). Analysing all indicators as possible predictors will provide us with information which of these factors may be most important for views of ageing.

We also assume that age and gender may play a role for these associations. Caregivers who are 65 years or older may be affected differently by aforementioned effects, than those aged 65 years and younger. Older age is often associated with an increased range of age-specific cues compared to younger age. In line with our findings from the previous study (Zwar et al. 2023) and with SST indicating socioemotional goals to be more important (Carstensen et al. 1999; Löckenhoff and Carstensen 2004), we assume that older caregivers may also benefit more from caregiver intensity regarding their personal views of ageing, at least in terms of care tasks and time than younger caregivers.

We also expect female caregivers to be affected more strongly by any associations. Women usually spend more hours on caregiving, provide more care tasks than men and experience higher caregiver burden (Pinquart and Sörensen 2006; Stanfors et al. 2019; Zygouri et al. 2021). Gender differences in views of ageing have been inconsistent; however, they indicate that women are usually more worried about old age and have a less favourable perspective on their ageing (Ayalon 2014; Bai 2014; Barrett and Von Rohr 2008). Thus, they may be more vulnerable to the activation of age stereotypes by age-specific cues such as informal caregiving and may therefore be affected more in terms of larger changes due to informal caregiving intensity than male caregivers.

## Method

### Sample

Data from wave 2014 and 2017 of the population-based German Ageing Survey from the German Centre for Gerontology were used (DZA, 2014, 2017). This is a cohort-sequential panel representing community-dwelling adults aged 40 years and older in Germany who were surveyed by means of an interview and an additional written questionnaire covering sensitive topics. The sample is extended every 6 years with a new sample drawn with a two-stage sampling method, stratified by age, gender and region. Earlier waves were excluded because they did not include all of the analysed variables (e.g. ATOA). We included all participants who provided informal care to a person with health-based care needs to an adult (caregivers for children or grandchildren were excluded; ‘Are there any persons who, due to their poor state of health, are looked after or cared for by you privately or on a voluntary basis, or for whom you provide regular help on a regular basis?’) and who had participated in interview and questionnaire (*N* = 2162). To analyse if changes in the predictors were associated with changes in the outcomes, we used Fixed Effects (FE) regression analyses, which includes only those participants for the estimation, who have experienced a change in the analysed variables (average treatment effect on the treated, ATET; Brüderl 2010). Written informed consent was provided by all participants. The criteria of the German Research Foundation for an ethics vote do not apply; therefore, an ethics vote was not needed and not applied for (Deutsche Forschungsgemeinschaft, 2010–2021).

### Variables

#### Main predictors

*Caregiving time* was measured as hours per week (‘How much time do you spend per week helping the person you support?’, Range: 0 to 168 h per week). Informal caregivers were asked ‘What help and support do you provide?’, in terms of household help, supervision and support, nursing care tasks or other care tasks. These care tasks summed up in a variable which provides information in how many of these areas caregivers provided support, resulting in our *range of care tasks* variable (Range: 0–4), thus, indicating the range or diversity of caregiving. *Caregiving burden* was measured by asking caregivers to consider all care and support they provide and evaluate how burdened they are by this performance (‘If you look at these aids or care services as a whole, how much of a burden do they place on you?’, Range: 1 not at all – 4 very much).

#### Outcomes

The German version of the subscale *attitude towards one’s own ageing* (ATOA) from the *Philadelphia Geriatric Center Morale Scale* (PGCMS; ‘The older I get, the worse everything becomes’, ‘Have same energy as last year’, ‘The older I get, the less useful I am’, ‘The older I get, life is better than expected’, ‘Now as happy as in younger years’, Range: 1–4) (Lawton 1975; Liang and Bollen 1983) was used. This is a reliable and well-established scale in research on perceptions of ageing (Cronbach’s *α* = 0.75–0.76; Kotter-Gruhn et al. 2009; Westerhof et al. 2014; Wurm et al. 2014). The items were poled so higher scores indicate a more positive perception of one’s own ageing and a mean score was calculated based on its 5 items (Range: 1–4; Beyer et al. 2015). *Subjective age* (SA) refers to how old people feel (‘Apart from your actual age: If you are to express it in years, how old do you feel?’). We treated all values three standard deviations above and below the sample mean score as outliers and excluded them, in line with procedures performed in previous research (Stephan et al. 2015; Weiss and Lang 2012). *Onset of old age* (OOA) was measured by asking people at what age they would consider someone as being old (At what age would you describe someone as old?). For this measure, we also excluded outliers, which were three standard deviations above and below the mean score.

#### Covariates

The caregiver’s sociodemographic background and health were measured. Chronological age was measured as continuous (beginning at 40 years) and dichotomous variable (middle-aged: < 65 years; older; ≥ 65 years). Gender included male and female as categories. Marital status (married and living together or separately vs. divorced, widowed or single) and employment status (employed vs. currently not employed, including retired and unemployed individuals) were measured with dichotomous variables. Health was measured in terms of self-rated health (Range: 1–5, higher values indicate worse health) and number of chronic illnesses (e.g., diabetes, cardiovascular disease; count score, Range: 0–11).

### Statistical analysis

We conducted FE regression analysis in this study (Brüderl 2010; Wooldridge 2010). With longitudinal data, unobserved heterogeneity can be differentiated into a time-constant and a time-varying (idiosyncratic) error. FE regression analysis are based on the assumption that the time-constant error is associated with the analysed variables and could severely bias the estimated parameters. Therefore, the method focuses only on time-varying factors and controls for all time-constant observed and unobserved variables (e.g., genetic disposition, gender). As a result, only time-varying covariates have to be controlled to fulfill the assumption that the idiosyncratic error is not associated with the analysed variables. This assumption is much weaker than assumptions of other panel analysis methods, such as Random or Mixed Effects methods, which rely on the assumption that the analysed variables are not associated with any error, time-constant or time-varying. Since this assumption is rarely fulfilled, estimates can be severely biased. The weaker assumptions of FE regression analysis are more likely to be fulfilled and enable the estimation of consistent parameters, i.e. enable to estimate the true (unbiased) value of the parameter. This is a major advantage in research with observational panel data. Results from Sargan–Hansen tests (Schaffer and Stillman 2016) support our decision to use FE regression analysis (results available upon request).

Since the method focuses only on time-varying factors, only participants who varied in the analysed variables are used for the estimation of the regression coefficients (ATET; Brüderl and Ludwig 2015). We used the *xtsum* command to check for variation in the continuous predictor variables. This is a command from the statistical software Stata that is used for longitudinal data (xt) and provides information on mean values and standard deviation. To reduce the risk of bias by serial autocorrelation and heteroscedasticity, we calculated robust standard errors (Cameron and Trivedi 2009). The sample of our analyses contained only very few missing values (below 5%); thus, listwise deletion was used (Allison 2001).

All models were adjusted for the caregiver’s health and sociodemographic data, except for gender and education. As time-constant variables they would be omitted during estimation of the FE regression analysis. Age and gender were used as moderators, i.e. we analysed interaction effects between dichotomized age, respective, gender, and the three caregiving intensity indicators, and both variables were used for stratification in further analyses. Age was dichotomized into two groups (middle-age: 40 to 64, old age: 65 years and older), to analyse if both groups of caregivers experience different associations between caregiving intensity and views of ageing. Both groups are representing a different group of caregivers as can be seen in the description of the sample in the supplementary data (Additional file 1: Table S1). All analyses were conducted with the statistical software Stata 16.0 (Stata Corp., College Station Texas). The level of significance was set at alpha 0.05.

**Table 1:** Results of fixed effects regression analysis

Variables	ATOA			Subjective age			Onset of old age		
	*b*	Robust SE	95% CI	*b*	Robust SE	95% CI	*b*	Robust SE	95% CI
Caregiver burden	− 0.03	(0.03)	[− 0.08; 0.03]	0.13	(0.53)	[− 0.91; 1.16]	− 0.74+	(0.45)	[− 1.62; 0.13]
Care time (h/week)	− 0.00+	(0.00)	[− 0.01; 0.00]	0.06*	(0.03)	[0.01; 0.11]	0.01	(0.03)	[− 0.04; 0.07]
Range of care tasks	0.07***	(0.02)	[0.03; 0.11]	− 0.26	(0.33)	[− 0.90; 0.38]	− 0.99**	(0.36)	[− 1.69; − 0.29]
Observations	2012			2046			1981		
*N*	1699			1726			1668		
*R*^2^	0.136			0.215			0.086		

Fixed Effects regression analysis adjusted for age (continuous variable), employment status, self-rated health and number of chronic diseases; unstandardized regression coefficients and robust standard errors are given. ATOA refers to attitudes towards own ageing (Range: 1–4), subjective age (Range: 5–110), onset of old age (Range: 50–100)

Level of significance: ***p < 0.001, **p < 0.01, *p < 0.05, +*p* < 0.10

## Results

### Description of the sample

The complete sample included 2162 informal caregivers (49.07% caring for parents, 23.59% for spouses or partners, and 26.97% for other related or non-related adults). They were on average aged 64.25 (± 10.25) years and 59.02% were female, and 95.42% had no migratory background. On average, they provided eleven hours of care per week (± 18.62) and were involved in 2.41 care task areas, primarily in *supervision and support* (83.02%). Level of burden was moderate (*M* = 2.14, SD =  ± 0.86). SA was on average 56.29 (± 11.76) years, ATOA was *M* = 3.00 (SD =  ± 0.53) and OOA was perceived at 75.10 (± 8.16) years of age. Further information on the complete and the subsamples are given in the Additional file 1: Table S1.

### Results of analysing the association between caregiving intensity and views of ageing

Using the complete sample (Table 1), FE regression analysis indicated a significant association between care time and increased SA (*b* = 0.06, *p* < 0.05). The number of care tasks was significantly associated with increased ATOA (*b* = 0.07, *p* < 0.001) and an earlier onset of old age (*b* = − 0.99, *p* < 0.01). No significant associations were found between caregiver burden and ATOA (*b* = − 0.03, *p* = 0.34), SA (*b* = 0.13, *p* = 0.81) and OOA (*b* = − 0.74, *p* < 0.10). No significant associations were found between care time and ATOA (*b* = − 0.00, *p* < 0.10) and OOA (*b* = 0.01, *p* = 0.67), and there was also no significant association between care tasks and SA (*b* = − 0.26, *p* = 0.43).

Moderator analyses with age indicated a significant interaction effect between age and burden (*b* = 0.13, *p* < 0.05) for the outcome ATOA (Table 2). The other interaction effects of the models analysing the outcome ATOA were not significant (care time × age: *b* = 0.00, *p* = 0.90; care tasks × age: *b* = − 0.01, *p* = 0.82). The interaction effects in analysis with the outcome SA (care time × age: *b* = − 0.11, *p* = 0.28; care tasks × age: *b* = − 0.91, *p* = 0.17; care burden × age: *b* = − 0.78, *p* = 0.45) and OOA (care time × age: *b* = 0.05, *p* = 0.51; care tasks × age: *b* = 0.19, *p* = 0.79; care burden × age: *b* = −0.01, *p* = 0.99) were not significant either. In additional stratified analyses (Additional file 1: Table S2), burden was associated with less positive ATOA among middle-aged caregivers (*b* = − 0.08, *p* < 0.10) and more positive ATOA among older caregivers (*b* = 0.03, *p* = 0.44), both non-significant associations. The number of care tasks was significantly associated with more positive ATOA (*b* = 0.08, *p* < 0.01) and earlier onset of old age (*b* = − 1.24, *p* < 0.05) among middle-aged informal caregivers. Among older caregivers, care time was significantly associated with less positive ATOA (*b* = − 0.00, *p* < 0.05) and higher SA (*b* = 0.05, *p* < 0.05), while care tasks were significantly associated with more positive ATOA (*b* = 0.08, *p* < 0.01). For further information on the stratified analyses, see Additional file 1: Table S2.

**Table 2:** Results of fixed effects regression analysis with age (model 1, 2, and 3) and gender (model 4, 5, and 6) as moderators

Variables	1			2			3			4			5			6		
	ATOA			Subjective age			Onset of old age			ATOA			Subjective age			Onset of old age		
	b	Robust SE	95% CI	b	Robust SE	95% CI	b	Robust SE	95% CI	b	Robust SE	95% CI	b	Robust SE	95% CI	b	Robust SE	95% CI
Caregiver burden	− 0.08+	(0.04)	[− 0.17; 0.00]	0.37	(0.72)	[− 1.05; 1.79]	− 0.69	(0.63)	[− 1.93; 0.55]	− 0.03	(0.05)	[− 0.13; 0.07]	0.00	(0.86)	[− 1.69; 1.69]	− 0.76	(0.70)	[− 2.13;0.61]
Care time (hours/week)	− 0.00	(0.00)	[− 0.01; 0.00]	0.17	(0.10)	[− 0.04; 0.37]	− 0.04	(0.07)	[− 0.18; 0.11]	0.00	(0.00)	[− 0.00; 0.00]	0.08+	(0.04)	[− 0.00; 0.17]	− 0.02	(0.04)	[− 0.10;0.06]
Range of care tasks	0.08**	(0.03)	[0.02; 0.14]	− 0.01	(0.40)	[− 0.79; 0.78]	− 1.01*	(0.47)	[− 1.93; − 0.09]	0.05	(0.04)	[− 0.03; 0.13]	1.00	(0.63)	[− 0.24; 2.25]	− 0.72	(0.62)	[− 1.93;0.50]
Alter	− 0.01	(0.01)	[− 0.02; 0.01]	1.15***	(0.15)	[0.86; 1.44]	0.44***	(0.13)	[0.19; 0.70]	− 0.01	(0.01)	[− 0.03; 0.01]	1.10***	(0.15)	[0.81; 1.38]	0.43***	(0.13)	[0.18;0.67]
Binary age (ref. < 65 years)	− 0.37*	(0.16)	[− 0.68; − 0.06]	3.94	(2.99)	[− 1.93; 9.81]	− 1.68	(2.57)	[− 6.72; 3.36]	–	–	–	–	–	–	–	–	–
Gender	–	–	–	–	–	–	–	–	–	[omitted]	[omitted]	[omitted]	[omitted]	[omitted]	[omitted]	[omitted]	[omitted]	[omitted]
Age (ref. < 65 years) × burden	0.13*	(0.06)	[0.02; 0.24]	− 0.78	(1.02)	[− 2.78; 1.23]	− 0.01	(0.88)	[− 1.74; 1.72]	–	–	–	–	–	–	–	–	–
Age (ref. < 65 years) × care time	0.00	(0.00)	[− 0.01; 0.01]	− 0.11	(0.10)	[− 0.32; 0.09]	0.05	(0.08)	[− 0.10; 0.20]	–	–	–	–					
Age (ref. < 65 years) × care tasks	− 0.01	(0.04)	[− 0.09; 0.07]	− 0.91	(0.67)	[− 2.23; 0.40]	0.19	(0.69)	[− 1.17; 1.54]	–	–	–	–	–	–	–	–	–
Gender (ref. Male) × burden	–	–	–	–	–	–	–	–		0.00	(0.06)	[− 0.12; 0.12]	0.09	(1.04)	[− 1.95;2.12]	0.21	(0.92)	[− 1.60;2.01]
Gender (ref. male) × care time	–	–	–	–	–	–	–	–		− 0.01*	(0.00)	[− 0.01; − 0.00]	− 0.04	(0.05)	[− 0.14;0.06]	0.05	(0.05)	[− 0.05;0.15]
Gender (ref. male) × care tasks	–	–	–	–	–	–	–	–		0.04	(0.05)	[− 0.05; 0.14]	− 1.82*	(0.74)	[− 3.26;− 0.38]	− 0.43	(0.76)	[− 1.92;1.06]
Observations	2012			2046			1981			2012			2046			1981		
*N*	1699			1726			1668			1699			1726			1668		
*R*^2^_within_	0.156			0.237			0.090			0.147			0.234			0.093		

Fixed Effects regression analysis adjusted for age (continuous variable), employment status, self-rated health and number of chronic diseases, gender was omitted from the analysis due to being time-constant; unstandardized regression coefficients and robust standard errors are given. ATOA refers to attitudes towards own ageing (Range: 1–4), subjective age (Range: 5–110), onset of old age (Range: 50–100)

Level of significance: ***p < 0.001, **p < 0.01, *p < 0.05, +*p* < 0.10

Moderator analyses with gender as moderator (Table 2) indicated a significant interaction effect between gender and care time (*b* = − 0.01, *p* < 0.05) for the outcome ATOA and between gender and care tasks (*b* = − 1.82, *p* < 0.05) for SA. The other interaction effects for outcome ATOA (care tasks × gender: *b* = 0.04, *p* = 36, care burden × gender: *b* = 0.00, *p* = 0.97), SA (care time × gender: *b* = − 0.04, *p* = 0.43; care burden × gender: *b* = 0.09, *p* = 0.93) and OOA (care time × gender: *b* = 0.05, *p* = 0.30; care tasks × gender: *b* = −0.43, *p* = 0.57; care burden × gender: *b* = 0.21, *p* = 0.82) were not significant. In additional stratified analysis (Additional file 1: Table S3), we found a significant associations between caregiving time (*b* = − 0.01, *p* < 0.001) and ATOA among female caregivers but not among male caregivers. Among female caregivers, we also found significant associations between caregiving tasks (*b* = 0.09, *p* < 0.001) and more positive ATOA, but not among male caregivers. Further analysis indicated significant associations between care tasks and lower SA (*b* = − 0.77, *p* < 0.05) and earlier OOA (*b* = − 1.16, *p* < 0.01) among female caregivers. Among male caregivers, caregiving time was significantly associated with higher SA (*b* = 0.09, *p* < 0.05), while the association between caregiving tasks and SA (*b* = 1.11, *p* < 0.10) was non-significant. For further information on these stratified analyses, see Additional file 1: Table S3.

Sensitivity analyses with type of care tasks and a discrepancy score of subjective age were conducted and can be found in the Additional file 1: Tables S4 and S5.

## Discussion

This study explored if specific aspects of the care situation could affect informal caregivers’ views of ageing and if this differed as a function of caregiver’s age and gender. To answer these research questions, the number of caregiving hours, range of care tasks and level of care burden were analysed in association with ATOA, SA, and OOA. Findings indicate that all three aspects of caregiving were associated with views of ageing in different ways. Whether they were positively or negatively associated varied with the age and gender of the caregiver.

Our findings partially confirm our expectations and add to previous findings (Loi et al. 2015; Luchesi et al. 2016; Zwar et al. 2022) by showing that specific aspects of the caregiving performance are associated with views of ageing in unique ways. SA was higher among informal caregivers with increasing hours of care per week. Also, the perception of age worsened as indicated by an earlier OOA in association with caregivers providing a broader range of care tasks. This could be because a broader range of care tasks likely reflect a broader level of care needs of the care recipient. Thus, more care intensity seems to bring one’s own age, closeness to old age, and age-related associations to the forefront of one’s mind. This negative change of views of ageing, in particular of SA, may endanger their health and well-being as indicated by previous findings (Alonso Debreczeni and Bailey 2020; Kotter-Gruhn et al. 2009; Westerhof et al. 2014).

However, a broader range of care tasks performed by caregivers was associated with more positive ATOA. Providing more diverse care tasks likely indicates a broader range of care needs of the care recipient but it may also highlight the caregivers own diverse abilities and therefore improve the perceptions of their own ageing process. Sensitivity analyses showed that the type of care task is also of relevance, namely household help is connected with more positive ATOA while nursing care tasks, i.e. personal care, was associated with earlier OOA. Further research on this and further care tasks is recommended.

### The significance of the caregivers’ age and gender

Aforementioned associations differed among caregivers based on their chronological age and gender. Burden, which was not directly associated with the outcomes, was associated with the caregiver’s ATOA as a function of chronological age. This perception of one’s own ageing worsened significantly more among middle-aged caregivers than among older caregivers with increasing burden. As an indicator of stress (Graessel et al. 2014), higher burden indicates more difficulties and a more negative evaluation of one’s ability to cope with caregiving, which could strengthen the salience of age-related cues and activate associated stereotypes of ageing (Levy 2009). These could highlight the caregiver’s own age-related limits and raise further concerns about their current and future ageing process. However, older caregivers may focus more on emotionally relevant goals in line with SST (Carstensen et al. 1999; Löckenhoff & Carstensen 2004). Our findings are in line with this. The positive aspects of caregiving, such as strengthening the relationship with the person in need of care, seemed to be more important for the evaluation of their own ageing than the burden of caregiving and prevented a worsening of ATOA. Further research on this is recommended.

In the stratified analysis we found further significant associations among the two age groups. Since they did not differ significantly (no significant interaction effects), these findings have to be interpreted with caution. Still, they provide further interesting insights. More diversity of care tasks improved ATOA while resulting in an earlier OOA among middle-aged caregivers. As explained before, this variety in care provides a more intense confrontation with possible age-related factors and therefore worsens the views of old age in general. However, the variety of care tasks can also provide a more nuanced contrast between one's own abilities and that of the cared-for, therefore resulting in a more positive evaluation of one’s own ageing (in terms of ATOA).

Among older caregivers, more diversity of care tasks had only a beneficial effect on the perception of their own ageing process (ATOA). This group may already be aware of difficulties that can occur with older age. Performing a broad range of care tasks may thus primarily highlight their own skills and actually negate many age stereotypes on diminished abilities and functions (Chasteen and Cary 2015). However, more caregiving time still worsened ATOA. This highlights that qualitative and quantitative aspects of caregiving intensity can have different consequences and should be analysed separately as done in this study.

Gender was also a significant factor for these associations. First, our findings pointed out that female caregivers were affected more in their views of ageing than male caregivers when aspects of the caregiving situation changed. This confirmed our expectations. Second, findings indicate that the pattern of change was more complex among women than among men.

Male caregivers perceived themselves as older (SA) when providing more hours of care and a broader range of care tasks. While they only differed significantly from women in the latter, these findings indicate that, for men, more caregiving in any form (time or tasks, i.e. quantitative and qualitative intensity) seems to be negatively affecting their views of ageing. In previous research male caregivers often reported difficulties with caregiving and having to learn new skills, such as cooking, cleaning and personal care (Russell 2007). They were also less likely to be involved in personal care (Pinquart and Sörensen 2006; Zygouri et al. 2021). More diversity of tasks may thus not provide more variety and highlight one’s own abilities, as found among female caregivers. Instead, it may only increase the challenge of caregiving and feelings of being overwhelmed, as previous findings indicated, resulting in worse views of ageing.

In contrast, among women, diversity of care improved the perceptions of one’s own ageing but worsened the views of ageing in general (OOA). Also, more caregiving hours worsened the perception of their own age, though it was their attitudes which changed and not their SA as found among men. Thus, while men feel older, women judge their own age and associated abilities more negatively. In sum, qualitative and quantitative aspects of care intensity have different effects on women and men. While men experience negative effects from both, women experience negative effects but can also benefit in particular from the qualitative aspect of care intensity, i.e. the diversity of care regarding their personal views of ageing.

### Limitations and advantages of the study

A few limitations of the study need to be discussed. Based on the range of our outcome, the changes we observed (i.e. the regression coefficients) were mostly small. Still, the findings provide evidence for the significance of caregiving to views of ageing. We measured burden with a single item construct. Further research is recommended which uses an instrument which allows for a more detailed assessment of the caregiving burden. Reverse causality cannot be excluded with the FE regression models. Also, panel attrition occurred (follow-up rates: 2014 38%, 2017 63%). However, this occurred due to age, gender, education and health (Schiel et al. 2018), which were all controlled explicitly or implicitly in our analysis. Additionally, the use of FE regression analysis has the advantage of accounting as well for all other unobserved time-constant variables which may be responsible for panel attrition or may be associated with the analysed variables (Brüderl 2010; Wooldridge 2010). Thus, the study has various advantages and can provide a good basis for future research and practical implications for influencing positive views of ageing positively among different groups of informal caregivers. It is the first study which analyses these associations with a longitudinal design and well-established instruments on views of ageing (such as PGCMS). The large population-based panel sample and the use of FE regression analysis are major advantages, which allow to significantly reduce the danger of bias by unobserved heterogeneity and enable consistent estimates. Also, the findings add to existing theoretical frameworks on views of ageing and highlight the significance of sociodemographic factors.

## Conclusion

In sum, this study’s findings provide new insight into views of ageing among informal caregivers, as well as age and gender differences, which highlight the need for different strategies to modify care performance to prevent a deterioration of views of ageing.

The findings show that informal caregivers could benefit in terms of better personal views of ageing from a reduction of the hours of care. Thus, sufficient and affordable professional care services are needed to ease the strain on caregivers and prevent negative changes in their views of ageing. Additionally, care performance should be modified in terms of reducing the hours but not the care tasks, since diversity of care tasks was associated with more positive attitudes towards their own ageing. For example, taking turns with professional care providers, such as using day or night care services, could be helpful. Also, integrating more professional care services to facilitate caregivers in carrying out a broad array of tasks could be helpful.

As further analyses pointed out, these suggestions should be adapted based on gender and age of informal caregivers. Based on our findings, we recommend to improve opportunities for diversity in care in all age groups of caregivers while focusing on a reduction of caregiving hours specifically for older caregivers. Quantitative caregiving intensity seems to be particularly problematic for this group’s views of ageing. In middle-aged caregiver groups, decreasing burden would be helpful for the perception of their own ageing process. This could be achieved, for example, by training caregivers in a broader range of coping strategies.

Also, male and female caregivers would benefit from decreasing the number of care hours per week, as mentioned above. Since enabling diversity in care tasks seems to be only helpful for female caregivers’ views of ageing, reduction of care hours should not be achieved by reducing the range of care tasks among female caregivers and leaving them to provide, for example, only personal care, which is often the care task female caregivers are mainly involved in (Pinquart and Sörensen 2006; Stanfors et al. 2019; Zygouri et al. 2021). Instead, designing care support that reduces intensity not diversity, especially among older and female caregivers, is recommended, to foster more positive personal views of ageing.

### Supplementary Information

Below is the link to the electronic supplementary material.

**Additional file 1.**
