# Supplementary material for: Caregiving intensity and its association with subjective views of ageing among informal caregivers with different sociodemographic background: a longitudinal analysis from Germany
Source: Eur J Ageing. 2024 Jan 13;21(1):4. doi: 10.1007/s10433-023-00797-4 (PMC10787706; doi:10.1007/s10433-023-00797-4)
Supplement: Supplementary file 1 — Additional file 1. [file 10433_2023_797_MOESM1_ESM.docx]

**Supplementary Material for the Manuscript *Caregiving intensity and its association with subjective views of ageing among informal caregivers with different sociodemographic background: a longitudinal analysis from Germany***

Table A1

Descriptive statistics for the pooled sample (2014 and 2017; N(%), M(SD))

|  | **Complete sample** | **< 65 years** | **≥ 65 years** | ***p*** | **male** | **female** | ***p*** |
| --- | --- | --- | --- | --- | --- | --- | --- |
| Observations | 2,162 | 1,150 | 1,012 |  | 886 | 1,276 |  |
| Age (years) | 64.25 (10.25) | 56.25 (5.46) | 73.34 (5.91) |  | 65.63 (10.51) | 63.30 (9.96) | <.001 |
| Gender (%) |  |  |  | <.001 | - | - |  |
| - male | 886 (40.98) | 426 (37.04) | 460 (45.45) |  |  |  |  |
| - female | 1,276 (59.02) | 724 (62.96) | 552 (54.55) |  |  |  |  |
| Education (ISCED) |  |  |  | <.001 |  |  | <.001 |
| - low | 81 (3.75) | 22 (1.91) | 59 (5.83) |  | 17 (1.92) | 64 (5.02) |  |
| - middle | 1,083 (50.09) | 581 (50.52) | 502 (49.60) |  | 391 (44.13) | 692 (54.23) |  |
| - high | 997 (46.11) | 547 (47.57) | 450 (44.47) |  | 478 (53.95) | 519 (40.67) |  |
| Relationship to care recipient (primary care provision) (%) |  |  |  | <.001 |  |  | <.001 |
| - parent-(in-law) | 1,061 (49.07) | 852 (74.09) | 209 (20.65) |  | 411 (46.39) | 650 (50.94) |  |
| - (ex-)partner/spouse | 510 (23.59) | 80  (6.96) | 430 (42.49) |  | 257 (29.01) | 253 (19.83) |  |
| - Other relatives, friends, neighbors or other acquaintances | 583 (26.97) | 217 (18.87) | 366 (36.17) |  | 213 (24.04) | 370 (29.00) |  |
| Marital status (%) |  |  |  | n.s. |  |  | <.001 |
| - married, living together or separately | 1,648 (76.23) | 875 (76.09) | 773 (76.38) |  | 724 (81.72) | 924 (72.41) |  |
| - divorced/ widowed/ single | 512 (23.68) | 274 (23.83) | 238 (23.52) |  | 162 (18.28) | 350 (27.43) |  |
| Employment status (%) |  |  |  | <.001 |  |  | <.05 |
| - employed | 852 (39.41) | 839 (72.96) | 13 (1.28) |  | 323 (36.46) | 529 (41.46) |  |
| - retired / unemployed | 1,309 (60.55) | 310 (26.96) | 999 (98.72) |  | 563 (63.54) | 746 (58.46) |  |
| Migration background |  |  |  | n.s. |  |  | n.s. |
| - no migratory background | 2,063 (95.42) | 1,089 (94.70) | 974 (96.25) |  | 851 (96.05) | 1,212 (94.98) |  |
| - migratory background and migratory experiences | 87 (4.02) | 56 (4.87) | 31 (3.06) |  | 33 (3.72) | 54  (4.23) |  |
| - migratory background without migratory experiences | 9 (0.42) | 4 (0.35) | 5 (0.49) |  | 2  (0.23) | 7  (0.55) |  |
| Self-rated health | 2.43 (.78) | 2.39 (.79) | 2.48 (.77) | <.001 | 2.47 (.79) | 2.40 (.78) | <.05 |
| Number of chronic illnesses | 2.55 (1.87) | 2.17 (1.77) | 2.97 (1.90) | n.s. | 2.57 (1.90) | 2.53 (1.86) | <.01 |
| Care time | 10.78 (18.62) | 8.07 (12.47) | 13.96 (23.51) | <.001 | 10.17 (18.01) | 11.20 (19.02) | n.s. |
| Care Burden | 2.14 (.86) | 2.17 (.82) | 2.11 (.90) | n.s. | 2.03 (.81) | 2.22 (.88) | <.001 |
| Care tasks |  |  |  |  |  |  |  |
| - number of care tasks | 2.41 (1.01) | 2.44 (.95) | 2.37 (1.07) | <.05 | 2.31 (.99) | 2.48 (1.01) | <.001 |
| - help in household (%) | 1,389 (64.25) | 781 (67.91) | 608 (60.08) | <.001 | 546 (61.63) | 843 (66.07) | <.05 |
| - supervision and support (%) | 1,795 (83.02) | 955 (83.04) | 840 (83.00) | n.s. | 715 (80.70) | 1,080 (84.64) | <.05 |
| - nursing care tasks (%) | 563 (26.04) | 257 (22.35) | 306 (30.24) | <.001 | 183 (20.65) | 380 (29.78) | <.001 |
| - other care tasks (%) | 1,459 (67.48) | 813 (70.70) | 646 (63.83) | <.01 | 601 (67.83) | 858 (67.24) | n.s. |
| Subjective age | 56.29 (11.76) | 49.30 (8.72) | 64.20 (9.54) | <.001 | 57.65 (11.65) | 55.34 (11.75) | <.001 |
| Attitudes towards own aging | 3.00 (.53) | 3.05 (.52) | 2.95 (.53) | <.001 | 2.98 (.54) | 3.02 (.52) | <.05 |
| Onset of old age | 75.10 (8.16) | 73.13 (7.92) | 77.33 (7.86) | <.001 | 73.62 (8.59) | 76.12 (7.69) | <.001 |

*Note*. Frequency and percentage are given for the categorical variables (N(%)), mean and standard deviation for the continuous variables (M(SD)); the pooled sample includes all participants who provided information in 2014 and 2017 in interview and questionnaire. Self-rated health (Range: 1-5), number of chronic illnesses (Range: 0-11), Care time (h/week, Range: 0-168), Care Burden (Range: 1-4), care tasks (Range: 0-4), subjective age (Range: 5-110), attitudes towards own ageing (ATOA; Philadelphia Geriatric Center Morale Scale, PGCMS, Range: 1-4), onset of old age (Range: 50-100); education (ISCED, International Standard Classification of Education; - low 0-2 no completed vocational education; middle 3-4 completed in-company or vocational school education or higher general school leaving certificate without completed vocational training; high 5-6 completed further education (vocational school, master school, technical school, vocational or technical academy) and respondents with a degree (university, university of applied sciences)).

Table A2

Results of Fixed Effects regression analysis stratified by age (<65 years vs. ≥65 years)

| Outcome | ATOA | | | | | | Subjective age | | | | | | Onset of old age | | | | | |
| --- | --- | --- | --- | --- | --- | --- | --- | --- | --- | --- | --- | --- | --- | --- | --- | --- | --- | --- |
| Age group | <65 years | | | ≥65 years | | | <65 years | | | ≥65 years | | | <65 years | | | ≥65 years | | |
|  | b | Robust SE | 95% CI | b | Robust SE | 95% CI | b | Robust SE | 95% CI | b | Robust SE | 95% CI | b | Robust SE | 95% CI | b | Robust SE | 95% CI |
| Caregiver burden | -0.08+ | (0.04) | [-0.16; 0.01] | 0.03 | (0.04) | [-0.05; 0.11] | 0.77 | (0.78) | [-0.76; 2.30] | -0.44 | (0.81) | [-2.03; 1.15] | -0.59 | (0.68) | [-1.92; 0.74] | -0.88 | (0.72) | [-2.29; 0.54] |
| Care time (hours/week) | -0.00 | (0.00) | [-0.01; 0.01] | -0.00* | (0.00) | [-0.01; -0.00] | 0.18 | (0.19) | [-0.19; 0.56] | 0.05* | (0.02) | [0.00; 0.10] | 0.02 | (0.13) | [-0.25; 0.28] | 0.01 | (0.03) | [-0.05; 0.08] |
| Number of care task areas | 0.08** | (0.03) | [0.02; 0.15] | 0.08** | (0.03) | [0.02; 0.14] | -0.17 | (0.49) | [-1.13; 0.80] | -1.08+ | (0.56) | [-2.19; 0.02] | -1.24* | (0.49) | [-2.21; -0.27] | -0.55 | (0.52) | [-1.57; 0.48] |
| Age | -0.00 | (0.01) | [-0.03; 0.02] | -0.01 | (0.01) | [-0.03; 0.02] | 1.01*** | (0.21) | [0.61; 1.42] | 1.34*** | (0.21) | [0.94; 1.75] | 0.46** | (0.17) | [0.13; 0.79] | 0.46* | (0.21) | [0.05; 0.87] |
| Employment status (ref. employed) |  |  |  |  |  |  |  |  |  |  |  |  |  |  |  |  |  |  |
| - retired / unemployed | 0.11 | (0.11) | [-0.09; 0.32] | 0.30*** | (0.06) | [0.19; 0.41] | 0.12 | (1.82) | [-3.46; 3.70] | -3.78 | (2.96) | [-9.59; 2.04] | 0.07 | (1.09) | [-2.06; 2.20] | -3.42 | (3.02) | [-9.35; 2.50] |
|  |  |  |  |  |  |  |  |  |  |  |  |  |  |  |  |  |  |  |
| Marital status (ref. married) |  |  |  |  |  |  |  |  |  |  |  |  |  |  |  |  |  |  |
| - divorced / widowed / single | -0.07 | (0.20) | [-0.46; 0.32] | -0.09 | (0.16) | [-0.42; 0.23] | 1.07 | (2.81) | [-4.44; 6.59] | 0.77 | (2.62) | [-4.37; 5.91] | -5.18*** | (0.69) | [-6.53; -3.84] | -3.43+ | (1.84) | [-7.04; 0.19] |
| Self-rated health | -0.11* | (0.05) | [-0.21; -0.00] | -0.15** | (0.05) | [-0.26; -0.04] | 0.34 | (0.71) | [-1.05; 1.73] | 0.50 | (0.86) | [-1.20; 2.19] | -0.79 | (0.54) | [-1.85; 0.27] | -0.11 | (0.85) | [-1.77; 1.56] |
| Number of chronic illnesses | -0.00 | (0.01) | [-0.03; 0.02] | -0.00 | (0.02) | [-0.04; 0.04] | -0.23 | (0.17) | [-0.57; 0.10] | 0.47+ | (0.26) | [-0.03; 0.97] | 0.41 | (0.32) | [-0.21; 1.03] | -0.31 | (0.42) | [-1.14; 0.51] |
| Constant | 3.48*** | (0.67) | [2.17; 4.80] | 3.37*** | (0.88) | [1.65; 5.09] | -11.12 | (10.71) | [-32.13; 9.89] | -30.52* | (14.49) | [-58.96; -2.08] | 53.80*** | (9.91) | [34.35; 73.26] | 51.96*** | (15.62) | [21.29; 82.62] |
| Observations | 1,094 |  |  | 918 |  |  | 1,101 |  |  | 945 |  |  | 1,066 |  |  | 915 |  |  |
| N | 931 |  |  | 798 |  |  | 938 |  |  | 817 |  |  | 907 |  |  | 789 |  |  |
| R² | 0.105 |  |  | 0.165 |  |  | 0.193 |  |  | 0.331 |  |  | 0.120 |  |  | 0.0673 |  |  |

*Note*. Fixed Effects regression analysis adjusted for employment status, self-rated health and number of chronic diseases, gender was omitted from the analysis due to being time-constant; unstandardized regression coefficients and robust standard errors are given. ATOA refers to attitudes towards own ageing (Range: 1-4), subjective age (Range: 5-110), onset of old age (Range: 50-100). Level of significance: *** p<0.001, ** p<0.01, * p<0.05, + p<0.10.

Table A3

Results of Fixed Effects regression analysis stratified by gender (male vs. female)

|  | ATOA | | | | | | Subjective age | | | | | | Onset of old age | | | | | |
| --- | --- | --- | --- | --- | --- | --- | --- | --- | --- | --- | --- | --- | --- | --- | --- | --- | --- | --- |
|  | male | | | female | | | male | | | female | | | male | | | female | | |
| VARIABLES | b | Robust SE | 95% CI | b | Robust SE | 95% CI | b | Robust SE | 95% CI | b | Robust SE | 95% CI | b | Robust SE | 95% CI | b | Robust SE | 95% CI |
|  |  |  |  |  |  |  |  |  |  |  |  |  |  |  |  |  |  |  |
| Caregiver burden | -0.04 | (0.05) | [-0.14; 0.06] | -0.03 | (0.03) | [-0.09; 0.04] | -0.18 | (0.87) | [-1.89; 1.53] | 0.14 | (0.62) | [-1.07; 1.35] | -0.84 | (0.69) | [-2.19; 0.52] | -0.46 | (0.59) | [-1.61; 0.70] |
| Caregiving time | 0.00 | (0.00) | [-0.00; 0.00] | **-0.01***** | (0.00) | [-0.01; - 0.00] | **0.09*** | (0.04) | [0.00; 0.18] | 0.04 | (0.03) | [-0.02; 0.10] | -0.02 | (0.04) | [-0.10; 0.05] | 0.04 | (0.03) | [-0.03; 0.10] |
| Caregiving tasks | 0.05 | (0.04) | [-0.03; 0.13] | **0.09***** | (0.02) | [0.04; 0.13] | 1.11+ | (0.65) | [-0.17; 2.38] | **-0.77*** | (0.37) | [-1.51; -0.04] | -0.57 | (0.59) | [-1.73; 0.60] | **-1.16**** | (0.43) | [-2.01; -0.31] |
| Age | -0.01 | (0.02) | [-0.04; 0.02] | -0.01 | (0.01) | [-0.03; 0.01] | 0.84** | (0.28) | [0.28; 1.40] | 1.23*** | (0.15) | [0.93; 1.53] | 0.59** | (0.21) | [0.18; 0.99] | 0.31+ | (0.16) | [-0.00; 0.62] |
| Employment status (ref. employed) |  |  |  |  |  |  |  |  |  |  |  |  |  |  |  |  |  |  |
| - retired / unemployed | 0.12 | (0.10) | [-0.07; 0.31] | 0.20* | (0.09) | [0.03; 0.38] | -2.04 | (2.34) | [-6.63; 2.54] | 0.99 | (1.17) | [-1.30; 3.29] | 1.81+ | (0.95) | [-0.05; 3.68] | -1.22 | (1.00) | [-3.18; 0.74] |
| Marital status (ref. married) |  |  |  |  |  |  |  |  |  |  |  |  |  |  |  |  |  |  |
| - divorced / widowed / single | -0.18 | (0.20) | [-0.58; 0.22] | -0.07 | (0.12) | [-0.31; 0.16] | -0.58 | (2.33) | [-5.15; 4.00] | 1.43 | (2.11) | [-2.71; 5.58] | -5.06* | (2.14) | [-9.26; -0.85] | -2.08 | (1.29) | [-4.60; 0.45] |
| Self-rated health | -0.12 | (0.08) | [-0.27; 0.04] | -0.16*** | (0.04) | [-0.24; -0.08] | 0.88 | (0.74) | [-0.57; 2.33] | 0.21 | (0.68) | [-1.12; 1.54] | -0.03 | (0.63) | [-1.26; 1.20] | -0.89 | (0.56) | [-1.99; 0.21] |
| Number of chronic illnesses | -0.00 | (0.03) | [-0.06; 0.05] | 0.00 | (0.01) | [-0.02; 0.02] | -0.42 | (0.33) | [-1.07; 0.22] | 0.20 | (0.16) | [-0.11; 0.51] | -0.80* | (0.38) | [-1.54; -0.06] | 0.42 | (0.27) | [-0.11; 0.95] |
| Constant | 3.67*** | (0.99) | [1.72; 5.62] | 3.87*** | (0.59) | [2.72; 5.03] | -0.43 | (17.02) | [-33.84; 32.97] | -23.22* | (9.25) | [-41.38; -5.07] | 40.31** | (13.55) | [13.70; 66.91] | 62.49*** | (10.19) | [42.49; 82.49] |
| Observations | 826 |  |  | 1,186 |  |  | 840 |  |  | 1,206 |  |  | 810 |  |  | 1,171 |  |  |
| N | 710 |  |  | 989 |  |  | 721 |  |  | 1,005 |  |  | 692 |  |  | 976 |  |  |
| R² | 0.0764 |  |  | 0.198 |  |  | 0.194 |  |  | 0.291 |  |  | 0.146 |  |  | 0.110 |  |  |

*Note*. Fixed Effects regression analysis adjusted for employment status, self-rated health and number of chronic diseases, gender was omitted from the analysis due to being time-constant; unstandardized regression coefficients and robust standard errors are given. ATOA refers to attitudes towards own ageing (Range: 1-4), subjective age (Range: 5-110), onset of old age (Range: 50-100). Level of significance: *** p<0.001, ** p<0.01, * p<0.05, + p<0.10.

Table A4

Analysis of main models with type of caregiving tasks instead of range of care tasks as predictor

|  |  | ATOA |  |  | Subjective age |  |  | Onset of old age |  |
| --- | --- | --- | --- | --- | --- | --- | --- | --- | --- |
| VARIABLES | b | Robust SE | 95% CI | b | Robust SE | 95% CI | b | Robust SE | 95% CI |
| Caregiver burden | -0.03 | (0.03) | [-0.09; 0.03] | 0.11 | (0.53) | [-0.93; 1.14] | -0.74+ | (0.44) | [-1.61; 0.12] |
| Care time (hours/week) | -0.00+ | (0.00) | [-0.01; 0.00] | 0.06* | (0.03) | [0.01; 0.12] | 0.02 | (0.03) | [-0.03; 0.07] |
| Care tasks |  |  |  |  |  |  |  |  |  |
| - household help | 0.14** | (0.05) | [0.05; 0.23] | -0.17 | (0.71) | [-1.57; 1.23] | -1.04+ | (0.61) | [-2.23; 0.15] |
| - supervision and support | 0.03 | (0.05) | [-0.07; 0.14] | 0.42 | (0.92) | [-1.39; 2.22] | -0.84 | (0.89) | [-2.59; 0.91] |
| - nursing care tasks | 0.05 | (0.05) | [-0.04; 0.15] | -0.54 | (0.71) | [-1.94; 0.86] | -2.37** | (0.77) | [-3.88; -0.86] |
| - other care tasks | 0.05 | (0.04) | [-0.02; 0.13] | -0.52 | (0.82) | [-2.13; 1.10] | 0.20 | (0.67) | [-1.12; 1.52] |
| Observations | 2,007 |  |  | 2,041 |  |  | 1,976 |  |  |
| N | 1,695 |  |  | 1,722 |  |  | 1,664 |  |  |
| R² | 0.142 |  |  | 0.217 |  |  | 0.104 |  |  |

*Note*. Fixed Effects regression analysis adjusted for age (continuous variable), employment status, self-rated health and number of chronic diseases; unstandardized regression coefficients and robust standard errors are given. ATOA refers to attitudes towards own ageing (Range: 1-4), subjective age (Range: 5-110), onset of old age (Range: 50-100). Level of significance: *** p<0.001, ** p<0.01, * p<0.05, + p<0.10.

Table A5

Analysis for outcome subjective age in terms of a discrepancy score

|  | b | Robust SE | 95% CI | b | Robust SE | 95% CI | b | Robust SE | 95% CI |
| --- | --- | --- | --- | --- | --- | --- | --- | --- | --- |
| Caregiver burden | 0.00 | (0.01) | [-0.01; 0.02] | 0.01 | (0.01) | [-0.02; 0.03] | -0.00 | (0.01) | [-0.03; 0.03] |
| Age (ref. <65 years) |  |  |  | 0.06 | (0.05) | [-0.03; 0.16] |  |  |  |
| caregiver burden x age (ref. <65 years |  |  |  | -0.01 | (0.02) | [-0.05; 0.02] |  |  |  |
| Care time | 0.00* | (0.00) | [0.00; 0.00] | 0.00 | (0.00) | [-0.00; 0.01] | 0.00+ | (0.00) | [-0.00; 0.00] |
| Care time x age |  |  |  | -0.00 | (0.00) | [-0.01; 0.00] |  |  |  |
| Range of care tasks | -0.00 | (0.01) | [-0.01; 0.01] | 0.00 | (0.01) | [-0.01; 0.01] | 0.02+ | (0.01) | [-0.00; 0.04] |
| Range of care tasks x age (ref. <65 years) |  |  |  | -0.01 | (0.01) | [-0.03; 0.01] |  |  |  |
| Gender (ref. male) |  |  |  |  |  |  | - |  | - |
| Care burden x gender (Ref. Male) |  |  |  |  |  |  | 0.00 | (0.02) | [-0.03; 0.04] |
| Care time x gender (Ref. Male) |  |  |  |  |  |  | -0.00 | (0.00) | [-0.00; 0.00] |
| Range of care tasks x gender (ref. male) |  |  |  |  |  |  | -0.03** | (0.01) | [-0.05; -0.01] |
| Constant | -0.38** | (0.14) | [-0.65; -0.10] | -0.44** | (0.14) | [-0.71; -0.17] | -0.37** | (0.14) | [-0.64; -0.09] |
|  |  |  |  |  |  |  |  |  |  |
| Observations | 2,046 |  |  | 2,046 |  |  | 2,046 |  |  |
| N | 1,726 |  |  | 1,726 |  |  | 1,726 |  |  |
| R² | 0.0398 |  |  | 0.0675 |  |  | 0.0650 |  |  |

*Note*. Outcome subjective age discrepancy score calculated as chronological age minus subjective age. Fixed Effects regression analysis adjusted for age (continuous variable), employment status, self-rated health and number of chronic diseases; unstandardized regression coefficients and robust standard errors are given. Level of significance: *** p<0.001, ** p<0.01, * p<0.05, + p<0.10.
